# Interplay between Siderophores and Colibactin Genotoxin Biosynthetic Pathways in *Escherichia coli*

**DOI:** 10.1371/journal.ppat.1003437

**Published:** 2013-07-11

**Authors:** Patricia Martin, Ingrid Marcq, Giuseppe Magistro, Marie Penary, Christophe Garcie, Delphine Payros, Michèle Boury, Maïwenn Olier, Jean-Philippe Nougayrède, Marc Audebert, Christian Chalut, Sören Schubert, Eric Oswald

**Affiliations:** 1 Inserm, UMR1043, Toulouse, France; 2 INRA, USC 1360, Toulouse, France; 3 CNRS, UMR5282, Toulouse, France; 4 Université de Toulouse, UPS, Centre de Physiopathologie de Toulouse Purpan (CPTP), Toulouse, France; 5 Jules Verne Picardie University, Medical school, Amiens, France; 6 Max von Pettenkofer-Institut für Hygiene und Medizinische Mikrobiologie, München, Germany; 7 CHU Toulouse, Hôpital Purpan, Service de bactériologie-Hygiène, Toulouse, France; 8 Neuro-gastroenterologie et Nutrition, UMR INRA/ENVT 1331, Toulouse, France; 9 INRA, UMR1331, Toxalim, Research Centre in Food Toxicology, Toulouse, France; 10 Université de Toulouse, UPS, IPBS, Toulouse, France; Faculté de Médecine Paris Descartes, site Necker, France

## Abstract

In *Escherichia coli*, the biosynthetic pathways of several small iron-scavenging molecules known as siderophores (enterobactin, salmochelins and yersiniabactin) and of a genotoxin (colibactin) are known to require a 4′-phosphopantetheinyl transferase (PPTase). Only two PPTases have been clearly identified: EntD and ClbA. The gene coding for EntD is part of the core genome of *E. coli*, whereas ClbA is encoded on the *pks* pathogenicity island which codes for colibactin. Interestingly, the *pks* island is physically associated with the high pathogenicity island (HPI) in a subset of highly virulent *E. coli* strains. The HPI carries the gene cluster required for yersiniabactin synthesis except for a gene coding its cognate PPTase. Here we investigated a potential interplay between the synthesis pathways leading to the production of siderophores and colibactin, through a functional interchangeability between EntD and ClbA. We demonstrated that ClbA could contribute to siderophores synthesis. Inactivation of both *entD* and *clbA* abolished the virulence of extra-intestinal pathogenic *E. coli* (ExPEC) in a mouse sepsis model, and the presence of either functional EntD or ClbA was required for the survival of ExPEC *in vivo*. This is the first report demonstrating a connection between multiple phosphopantetheinyl-requiring pathways leading to the biosynthesis of functionally distinct secondary metabolites in a given microorganism. Therefore, we hypothesize that the strict association of the *pks* island with HPI has been selected in highly virulent *E. coli* because ClbA is a promiscuous PPTase that can contribute to the synthesis of both the genotoxin and siderophores. The data highlight the complex regulatory interaction of various virulence features with different functions. The identification of key points of these networks is not only essential to the understanding of ExPEC virulence but also an attractive and promising target for the development of anti-virulence therapy strategies.

## Author Summary

The synthesis of numerous molecules involved in the virulence potential and fitness of pathogenic bacteria requires a particular enzyme family, *i.e.* phosphopantetheinyl transferases (PPTases). To date, the synthesis of a given bioactive metabolite was thought to require a specific PPTase. As PPTases are being investigated as promising targets for antibacterial development, we addressed the question of a possible functional interchangeability between PPTases in *Escherichia coli*. PPTases are known to be involved in the synthesis of low-molecular weight iron chelators (siderophores), and of a genotoxin named colibactin. Here we demonstrated interplay between the synthesis pathways leading to the production of siderophores and of colibactin. We showed that inactivation of both PPTases abolished the virulence of extra-intestinal pathogenic *E. coli* (ExPEC) in a mouse sepsis model. To our knowledge, this is the first demonstration of interplay between multiple PPTases-requiring pathways leading to the biosynthesis of functionally distinctive virulence factors, in a given microorganism. The extensive substrate specificity of PPTase ClbA could account for the co-selection and co-evolution of genomic islands encoding colibactin and yersiniabactin siderophore.

## Introduction

*Escherichia coli* is a normal resident of the lower-gut of humans and animals. Although usually a commensal, *E. coli* can be also a pathogen, associated with diarrheal disease and extra-intestinal infections [1], [2]. The majority of *E. coli* strains can be assigned to one of five main phylogenetic groups: A, B1, B2, D and E [3]. Strains of the distinct phylogenetic groups differ in their phenotypic and genotypic characteristics [4]–[6]. Extra-intestinal pathogenic *E. coli* (ExPEC), which display enhanced ability to cause infection outside the intestinal tract, carry specific genetic determinants or virulence factors that are clustered on different pathogenicity islands [7]. These virulence factors associated with extra-intestinal infections are nonrandomly distributed, and strains of the *E. coli* phylogenetic group B2 harbor the greatest frequency and diversity of virulence traits [8], [9].

As iron bioavailability is limited in the host, ExPEC are known to synthesize up to four types of siderophores involved in iron uptake: enterobactin, salmochelins, yersiniabactin and aerobactin [10], [11]. The biosynthesis of the first three requires a 4′-phosphopantetheinyl transferase (PPTase). These enzymes activate polyketide synthases (PKSs) and nonribosomal peptide synthetases (NRPSs) by catalyzing the transfer of a phosphopantetheinyl (P-pant) moiety from coenzyme A to conserved serine residues on PKSs and NRPSs [12], [13]. In organisms containing multiple P-pant-requiring pathways, each pathway generally involves a dedicated cognate PPTase [12]. In *E. coli*, the EntD PPTase is involved in the synthesis of enterobactin [14] and salmochelins, which are glycosylated forms of enterobactin [15]. The IroA locus responsible for salmochelins production is located either on a chromosomal pathogenicity island or on a transmissible plasmid [16]. Contrary to enterobactin, salmochelins are able to evade the mammalian innate immune response protein lipocalin 2 (siderocalin) and are therefore more potent virulence factors [17]. The other siderophore necessitating a PPTase is yersiniabactin. This siderophore is encoded by the high-pathogenicity island (HPI) that was acquired through horizontal transfer [18]. The HPI core region was detected in more than 70% of ExPEC isolated from blood cultures, urine samples and cerebrospinal fluid [19]. While yersiniabactin production in *Yersinia* requires the YbtD PPTase encoded outside the HPI [20], no gene homologous to *ybtD* has been identified in the genome of *E. coli* strains producing yersiniabactin. The PPTase committed to the synthesis of yersiniabactin in *E. coli* remains unknown.

We have shown that a number of *E. coli* strains from phylogenetic group B2 display also the *pks* island, which codes for the production of colibactin, a polyketide-non ribosomal peptide genotoxin [21]. Colibactin is known to induce DNA double-strand breaks, cell cycle arrest in G2-phase and megalocytosis in infected eukaryotic cells [21]. *E. coli* strains harboring the *pks* island can induce DNA damage in enterocytes *in vivo* and trigger genomic instability in mammalian cells [22]. In a rodent model of colon inflammation, colibactin was demonstrated to potentiate the development of colon cancer [23]. Surprisingly, colibactin is also required for the colonic anti-inflammatory properties of the probiotic *E. coli* strain Nissle 1917 [24]. The synthesis of colibactin requires a PPTase encoded by the *clbA* gene located on the *pks* island [21]. Epidemiological studies revealed that the majority (73.1%) of the colibactin-positive *E. coli* strains was clinical ExPEC and that the *pks* island was significantly associated with a highly virulent subset of ExPEC isolates [25]. Strikingly, an analysis of the prevalence of the colibactin island among Enterobacteriaceae revealed that the *pks* island was constantly associated with the yersiniabactin gene cluster [26].

In this work we investigated a potential interplay between the biosynthetic pathways leading to the production of siderophores and of the colibactin genotoxin, through a possible functional interchangeability between PPTases in *E. coli*. We demonstrated that ClbA can contribute to the synthesis of siderophores both *in vitro* and *in vivo*. We proved in a mouse model of sepsis that the presence of either functional EntD or ClbA is required to maintain full virulence of ExPEC. This evidenced the interconnection between pathways leading to the synthesis of distinct secondary metabolites, via the PPTase ClbA. Therefore, the strict association of the *pks* island with HPI could have been selected in highly virulent *E. coli* isolates because ClbA can contribute to the synthesis of both the genotoxin and yersiniabactin.

## Results

### The *pks* island does not code for the biosynthesis of a siderophore *in vitro*

**Figure 1:**
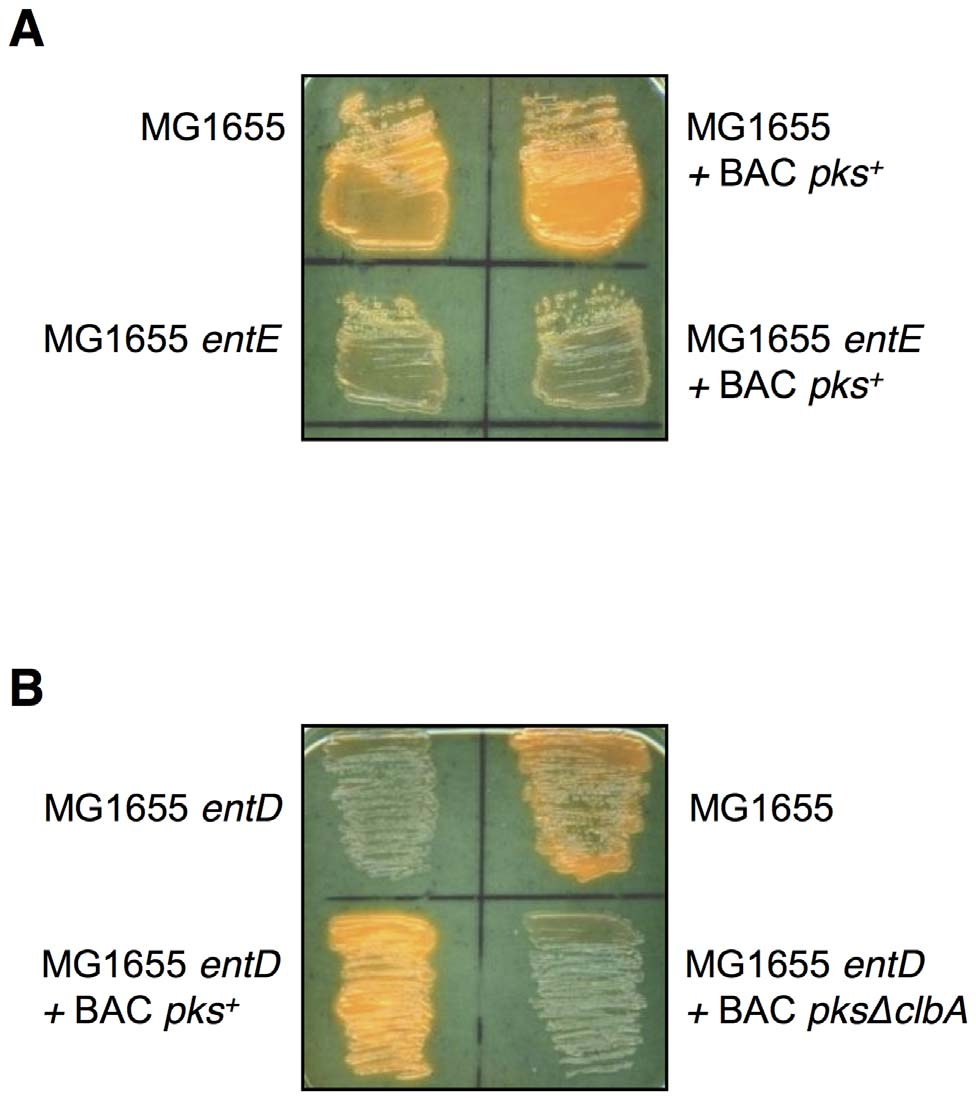
Siderophore production by *Escherichia coli* strain MG1655 and derivatives. Chrome azurol S (CAS) plates upon which the *E. coli* strain MG1655 and derivatives have been grown overnight. **A.** Wild type and *entE* derivatives of strain MG1655. **B.** Wild type and *entD* derivatives of strain MG1655. BAC *pks* ^+^ is a bacterial artificial chromosome (BAC) harboring the entire *pks* island. BAC *pksΔclbA* is a BAC harboring the entire *pks* island where the *clbA* gene was deleted. A yellow halo is produced around siderophore secreting bacteria.

Because colibactin and siderophores belong to the same family of chemical compounds, we investigated first whether the *pks* island could not only allow the production of a genotoxin, but also of a siderophore. The *entE* gene, that encodes the ligase component of synthase multienzyme complex necessary for the enterobactin biosynthesis, was inactivated in the enterobactin producer *E. coli* strain MG1655. The resulting MG1655 *entE* mutant strain was shown not to produce any siderophore, as detected on CAS plate (Fig. 1A). The wild type (WT) and *entE* derivative of strain MG1655 were transformed with the bacterial artificial chromosome (BAC) harboring the entire *pks* island (BAC *pks* ^+^). Both strains MG1655+BAC *pks^+^* and MG1655 *entE*+BAC *pks^+^* were shown to produce the genotoxin, as evidenced by the induction of double-strand breaks in eukaryotic cells (data not shown). The production of siderophore was qualitatively investigated in the resulting strains by plating on CAS plates (Fig. 1A). A yellow halo was not observed surrounding the bacterial colonies of strain MG1655 *entE*+BAC *pks^+^*. This showed that the *pks* island did not code for the biosynthesis of a siderophore.

### ClbA, the PPTase encoded on the *pks* island, can support the enterobactin siderophore synthesis *in vitro*

In order to test whether the ClbA PPTase was functionally capable of participating to the biosynthesis of enterobactin, the *entD* gene was disrupted in *E. coli* strain MG1655. The resulting MG1655 *entD* mutant strain was subsequently transformed with BAC *pks* ^+^ and with the BAC harboring the entire *pks* island where the *clbA* gene was deleted (BAC *pksΔclbA*). The production of siderophore was investigated by plating the resulting strains on CAS medium (Fig. 1B). This revealed that disruption of the *entD* gene in strain MG1655 resulted in the abrogation of the production of enterobactin (Fig. 1B). The introduction of the intact *pks* island in strain MG1655 *entD* restored the production of yellow pigmentation surrounding the colonies. This was not observed upon the introduction of the *pks* island disrupted for the *clbA* gene (Fig. 1B). Introduction of a functional plasmidic *clbA* gene in strain MG1655 *entD*+BAC *pksΔclbA* and in strain MG1655 *entD* restored the production of enterobactin (data not shown).

These data evidenced that the ClbA PPTase can contribute to the enterobactin siderophore synthesis *in vitro*.

### Both EntD and ClbA can support the yersiniabactin siderophore synthesis *in vitro*

Yersiniabactin is a siderophore the biosynthesis of which requires the PPTase YbtD in *Yersinia pestis* [20]. Although numerous *E. coli* strains were shown to produce yersiniabactin, an *in silico* analysis of the genome of all the *E. coli* strains available to date did not reveal any gene homologous to the *ybtD* gene.

**Figure 2:**
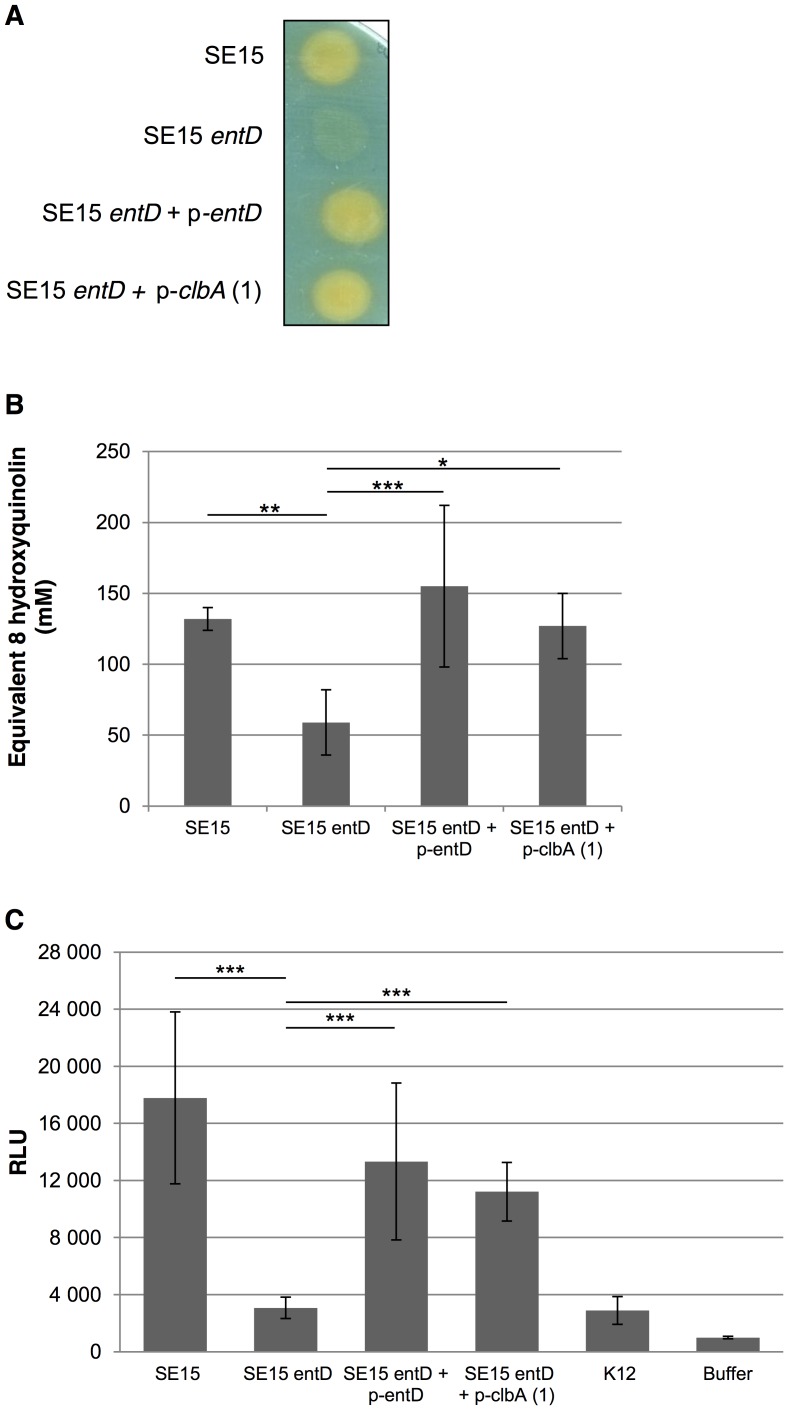
Both EntD and ClbA can support the yersiniabactin siderophore synthesis *in vitro*. Siderophore production by the enterobactin and yersiniabactin siderophores producer *Escherichia coli* strain SE15 and derivatives. **A.** Chrome azurol S (CAS) plate upon which *E. coli* strain SE15 and derivatives have been streaked for overnight growth. **B.** Quantification of total siderophore production in supernatants of *E. coli* strain SE15 and derivatives determined by the CAS assay. The data are the means and standard deviations of 5 independent experiments. **C.** Quantification of the yersiniabactin siderophore production in *E. coli* strain SE15 and derivatives. *E. coli* strains HB101, MG1655 and DH5α were used as negative controls (K12). RLU: relative light units. ***: *P*<0.001, **: *P*<0.01, *: *P*<0.05.

In order to test whether the ClbA PPTase was functionally proficient to participate to the biosynthesis of yersiniabactin, we analyzed the enterobactin and yersiniabactin producer *E. coli* strain SE15. The *entD* gene was disrupted in *E. coli* strain SE15. The resulting SE15 *entD* mutant was subsequently transformed with plasmids carrying wild type *entD* gene or *clbA* gene. The production of total siderophores was qualitatively (Fig. 2A) and quantitatively (Fig. 2B) investigated using the CAS assay. This revealed that disruption of the *entD* gene resulted in the abrogation of the production of siderophores in strain SE15 (Fig. 2A and 2B). As expected, complementation with *entD* gene restored the production of siderophores. Remarkably, complementation with *clbA* gene also resulted in the synthesis of siderophores (Fig. 2A and 2B). The synthesis of yersiniabactin was specifically quantified in the different SE15 derivatives (Fig. 2C). This revealed that in the *entD* mutant, the yersiniabactin biosynthesis was abolished. The introduction of *entD* or *clbA* genes in SE15 *entD* mutant strain resulted in the restoration of yersiniabactin production.

These data showed that in *E. coli* strain SE15, EntD is the PPTase dedicated to the synthesis of yersiniabactin. Moreover, the EntD function can be substituted by ClbA. This suggests that both EntD and ClbA are involved in the synthesis of yersiniabactin in *E. coli* strains producing endogenously EntD and ClbA.

### Colibactin synthesis cannot be sustained by EntD *in vitro*

**Figure 3:**
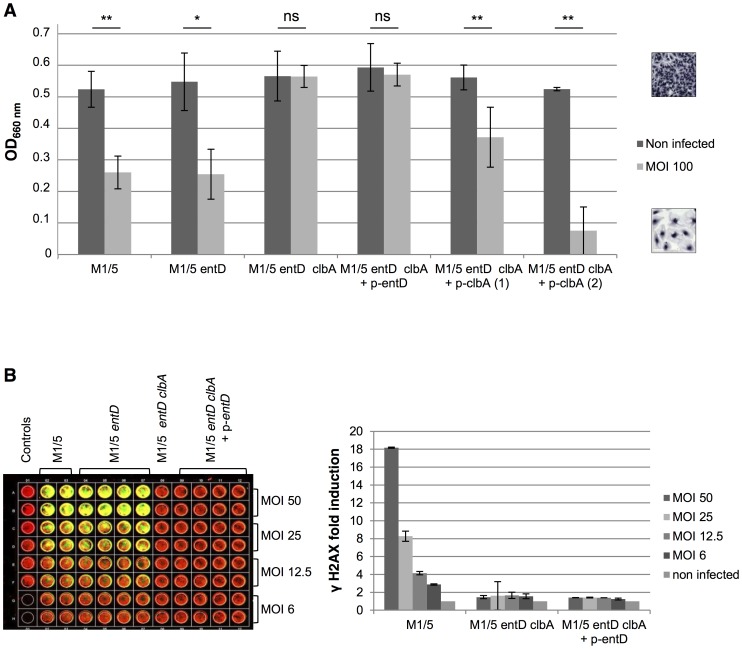
Colibactin synthesis cannot be sustained by EntD *in vitro*. Colibactin production by *Escherichia coli* strain M1/5 and derivatives determined by megalocytosis (**A**) and by quantification of DNA double strand breaks (**B**) in infected HeLa cells. **A.** Live *E. coli* wild type strain M1/5, mutants and complemented derivatives were added directly onto HeLa cells [multiplicity of infection (MOI) = 100], cocultivated for 4 h, then washed as described in Nougayrède et al. [21]. The cells were incubated for 72 h with gentamicin before protein staining with methylene blue. The quantification of staining was measured at OD 660 nm. **: *P*<0.01, *: *P*<0.05, ns: not significant. **B.** Quantification of DNA double strand breaks through the quantification of phosphorylated H2AX (γ-H2AX) using In Cell Western method [27]. HeLa cells were infected 4 h with strain M1/5 and derivatives [MOI = 50 to 6] fixed, and examined 8 h post infection for quantification of γ-H2AX.

As our data demonstrated that ClbA could complement EntD for the synthesis of enterobactin and yersiniabactin, we investigated whether EntD could rescue a *clbA* mutant for the production of colibactin. The *entD* gene was disrupted alone or in combination with the *clbA* gene in the colibactin producing *E. coli* strain M1/5. The M1/5 *entD clbA* double mutant was transformed with multicopy plasmids harboring wild type *entD* or *clbA* genes. The production of colibactin was quantified in the resulting strains through the quantification of megalocytic cells (Fig. 3A) and phosphorylation of H2AX histone (Fig. 3B) which correlate with DNA double strand breaks resulting from the genotoxic effect of colibactin [21], [22].

HeLa cells were infected with the different strains for 4 hours, fixed and stained with methylene blue in order to quantify the megalocytosis effect, as previously described [21]. This revealed that the megalocytosis effect observed with the M1/5 *entD* mutant strain was similar to the effect measured with the wild type M1/5 strain (Fig. 3A). Inactivation of the *clbA* gene in the M1/5 *entD* mutant abrogated the colibactin effect (Fig. 3A). Transformation of the M1/5 *entD clbA* mutant strain with plasmids carrying the functional wild type *clbA* gene resulted in the restoration of the megalocytosis. A partial complementation of the double mutation was observed with plasmid p-*clbA* (1) whereas the double mutant was fully complemented with p-*clbA* (2). The different copy number of the plasmids can account for the quantitative differences observed below. A complementation was not observed when the wild type *entD* gene was expressed from a multicopy plasmid in the double mutant (Fig. 3A).

Genotoxicity of colibactin [21] was also examined in HeLa cells using H2AX assay based on indirect DNA double strand break detection using In Cell Western (ICW) with infrared fluorescence for H2AX phosphorylation (γ-H2AX) quantification [27]. HeLa cells were infected with strains M1/5, M1/5 *entD*, M1/5 *entD clbA* or M1/5 *entD clbA* complemented with *entD*. Following the quantification of the γ-H2AX (green) and the DNA (red) signals (Fig. 3B), respectively, the fold induction of γ-H2AX per cell was calculated. This revealed a genotoxic dose–response depending on the multiplicity of infection (MOI, Fig. 3B). No difference of γ-H2AX per cell was observed between WT and *entD* mutant strains. Infection of HeLa cells with mutant M1/*5 entD clbA* did not induce phosphorylation of H2AX. Moreover, the introduction of the functional *entD* gene did not result in the generation of DNA double strand breaks in strain M1/5 *entD clbA* (Fig. 3B).

Altogether, these data evidenced that EntD does not contribute to the colibactin synthesis, even when highly expressed on a multicopy plasmid.

### Colibactin synthesis can be sustained by exogenous PPTases *in vitro*

**Figure 4:**
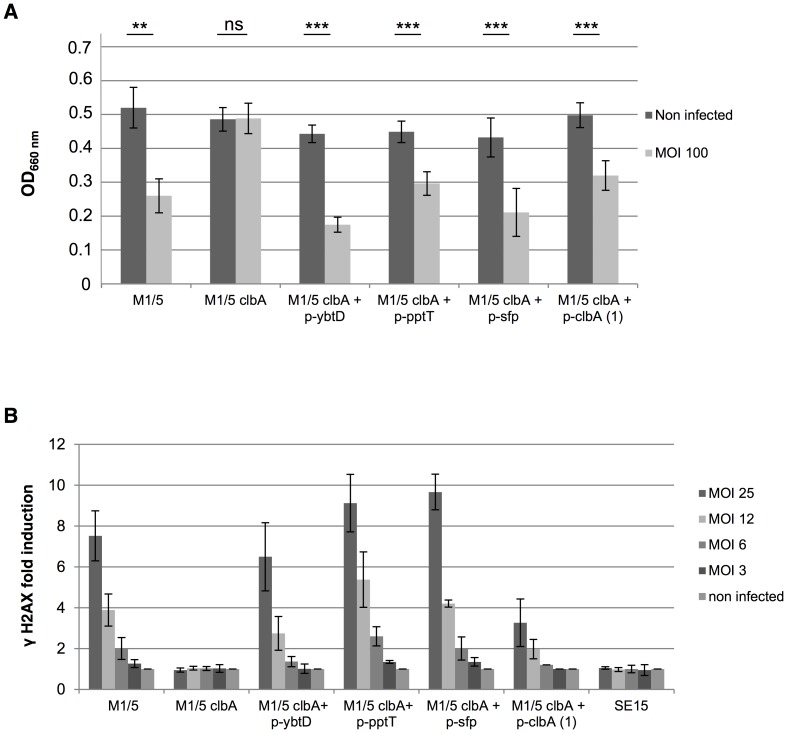
Colibactin synthesis can be sustained by exogenous PPTases *in vitro*. Colibactin production by *Escherichia coli* strain M1/5 and derivatives determined by megalocytosis (**A**) and by quantification of DNA double strand breaks (**B**), as in Fig. 3. ***: *P*<0.001, **: *P*<0.01, ns: not significant. *E. coli* strain SE15, which is devoid of colibactin locus, was used as a negative control. The *ybtD* gene encodes the YbtD PPTase in *Yersinia pestis*, the *pptT* gene the PptT PPTase in *Mycobacterium tuberculosis*, and the *sfp* gene the Sfp PPTase in *Bacillus subtilis*.

We then investigated whether other PPTases, originated from other bacterial species, could rescue a *clbA* mutant for the production of colibactin. The *clbA* gene was disrupted in *E. coli* strain M1/5. The M1/5 *clbA* mutant was transformed with plasmids harboring wild type *ybtD* gene that encodes the YbtD PPTase in *Yersinia pestis*, *pptT* gene the PptT PPTase in *Mycobacterium tuberculosis*, *sfp* gene the Sfp PPTase in *Bacillus subtilis*, and *clbA* gene. PptT is involved in biosynthesis of the mycobactin siderophore [28] and is essential for mycobacterial viability [29]. Sfp is required for production of the peptide antibiotic surfactin [30]. The production of colibactin was quantified in the resulting strains through the quantification of megalocytic cells (Fig. 4A) and phosphorylation of H2AX histone (Fig. 4B). This revealed that both the megalocytosis and the H2AX phosphorylation were restored in the *clbA* mutant upon introduction of *ybtD*, *pptT* and *sfp* genes.

These data evidenced that ClbA can be xeno-complemented for the colibactin synthesis.

### ClbA is more promiscuous in its substrate specificity than EntD

**Figure 5:**
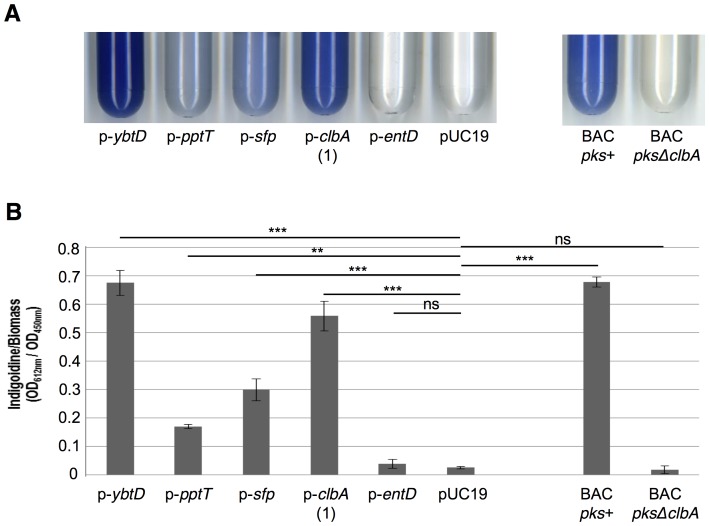
ClbA is more promiscuous in its substrate specificity than EntD. The synthesis of the single-module non-ribosomal peptide synthetase BpsA from *Streptomyces lavendulae* resulting in the production of indigoidine was qualitatively (**A**) and quantitatively (**B**) assessed as previously described [31], [51]. *E. coli* strain MG1655 Δ*entD*+p-*BpsA* was transformed with the plasmids p-*ybtD*, p-*pptT*, p-*sfp*, p-*clbA* (1), p-*entD* and pUC19 (left). *E. coli* strain MG1655 BAC *pks* ^+^ and MG1655 BAC *pksΔclbA* were transformed with p-*BpsA* (right). **A.** All the strains were grown overnight at 18°C in an auto-inducer medium. The cultures were left at room temperature so that the blue pigmentation (indigoidine) is produced. **B.** Quantification of indigoidine in the different strains. The data are the means and standard deviations of 3 independent experiments. ***: *P*<0.001, **: *P*<0.01, ns: not significant.

In order to confirm that EntD and ClbA have narrow and broad substrate-specificity, respectively, we investigated whether EntD and ClbA had the capacity to activate the carrier protein involved in a reporter biosynthetic pathway. When activated by a PPTase, the single-module non-ribosomal peptide synthetase BpsA from *Streptomyces lavendulae* synthesizes a colored product (indigoidine), from a single substrate (L-glutamine) [31]. Plasmid p-*BpsA* that encodes BspA was transformed into strain MG1655 *entD*. The resulting MG1655 Δ*entD*+p-*BspA* strain was subsequently transformed with plasmids carrying *ybtD*, *pptT*, *sfp*, *clbA*, or *entD* genes. In addition, *E. coli* strain MG1655 BAC *pks* ^+^ and MG1655 BAC *pksΔclbA* were transformed with p-*BpsA*. The resulting strains that carry both the NRPS and a functional PPTase were grown in auto-induction medium, as previously described [32]. A blue coloration was detectable in cultures after overnight incubation for all strains but strain MG1655 Δ*entD*+p-*BspA*+p-*entD* (Fig. 5A). A quantification of the indigoidine production was determined for all the strains (Fig. 5B). This confirmed that contrary to EntD, the PPTases YbtD, PptT, Sfp and ClbA were able to participate to the synthesis of the blue pigment.

This strengthens the fact that ClbA is more promiscuous in its substrate specificity than EntD in *E. coli*.

### Both EntD and ClbA must be inactivated to abolish virulence of ExPEC in a mouse model of sepsis

**Figure 6:**
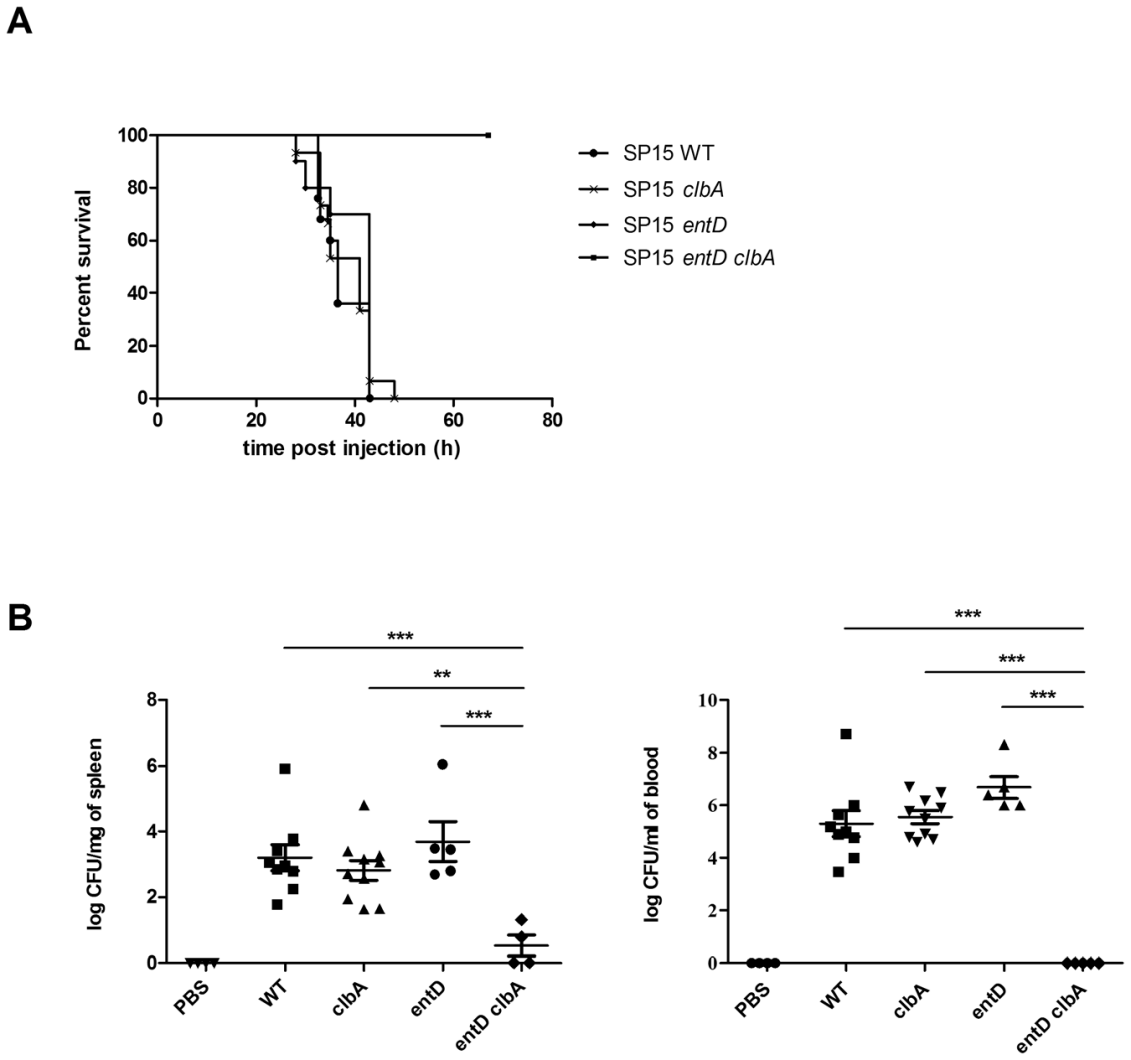
Both EntD and ClbA must be inactivated to abolish virulence of ExPEC. Mice underwent footpad injection with 10^8^ CFU of *E. coli* SP15 wild type strain or derivatives. **A.** The percentage of mice survival was monitored. 10 to 25 mice were used per group. **B.** 18 h post infection 4 to 10 mice per group were sacrificed. Bacteria were quantified in spleen and blood collected from each animal. For statistical analysis, two-factor ANOVA and Bonferroni's multiple comparison test was performed. ***: *P*<0.001, **: *P*<0.01.

In order to address the consequences, on the virulence of *E. coli*, of the cross talk between the synthesis pathways of colibactin and siderophores demonstrated *in vitro*, we investigated *E. coli* strain SP15, an extra-intestinal pathogenic *E. coli* strain (ExPEC) of serotype O18:K1:H7 isolated from neonatal meningitis, in a mouse model of sepsis. *E. coli* strain SP15 produces colibactin and four different siderophores (aerobactin, yersiniabactin, enterobactin and salmochelin). The *entD* or *clbA* genes were disrupted individually and in combination. The strains were injected individually into the mice footpad; and the mice survival was monitored (Fig. 6A). This revealed that all the strains but SP15 *entD clbA* induced 70% mortality within 40 hours after injection. In contrast, virulence of strain SP15 *entD clbA* was completely attenuated in this mouse model of sepsis (Fig. 6A).

The bacterial dissemination in the mice was analyzed (Fig. 6B). Mice were sacrificed 18 hours post injection with PBS, WT strain, single or double mutants. Spleens and blood samples were collected, and bacteria were quantified by plating on selective medium (Fig. 6B). We observed that in both spleen and blood of infected animals the bacterial loads were similar with all the strains, but strain SP15 *entD clbA*. No bacteria were recovered from spleen or blood of mice injected with the double mutant SP15 *entD clbA* (Fig. 6B).

This demonstrated that both EntD and ClbA must be inactivated to abolish virulence of ExPEC in a mouse model of sepsis.

### The presence of either functional EntD or ClbA is required to maintain full virulence of ExPEC in a mouse model of sepsis

**Figure 7:**
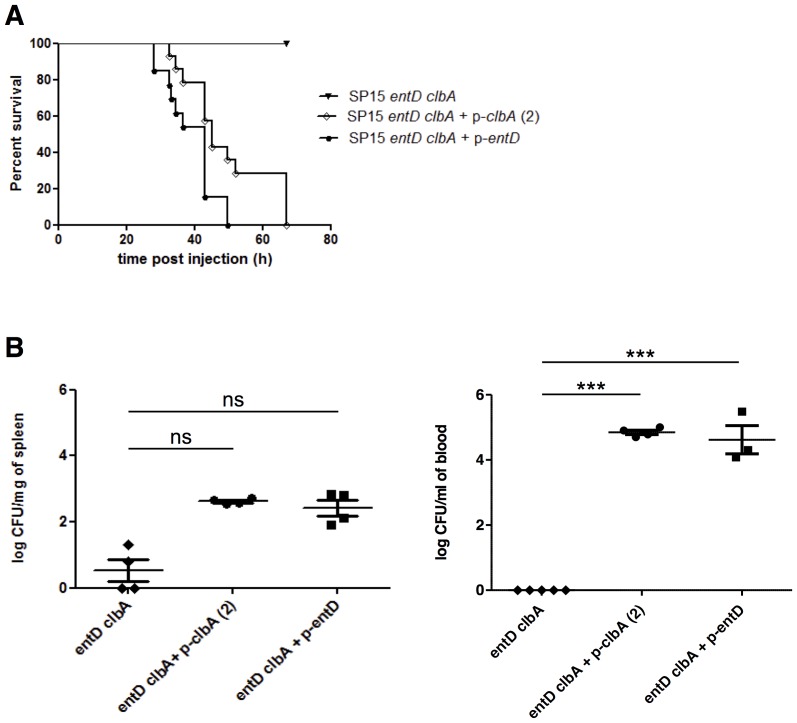
The presence of either EntD or ClbA is required to maintain full virulence of ExPEC. Mice underwent footpad injection with 10^8^ CFU of *E. coli* SP15 *entD clbA* strain and complemented derivatives. **A.** The percentage of mice survival was monitored. 10 to 25 mice were used per group. **B.** 18 h post infection 3 to 5 mice per group were sacrificed. Bacteria were quantified in spleen and blood collected from each animal. For statistical analysis, two-factor ANOVA and Bonferroni's multiple comparison test was performed. ***: *P*<0.001, ns: not significant.

In order to investigate the relative importance of EntD and ClbA in the virulence of *E. coli*, the SP15 *entD clbA* mutant strain was transformed with plasmids harboring *clbA* or *entD* functional genes. The resulting complemented strains were injected in mice (Fig. 7). This showed that complementation of strain SP15 *entD clbA* with either *clbA* or *entD* totally restored the virulence of the strain (Fig. 7A). A slight but statistically significant delay in survival kinetics was observed when strain SP15 *entD clbA* complemented with the *clbA* gene was used for the injections (Fig. 7A). The quantification of bacteria in spleen and blood of the infected animals was determined (Fig. 7B). This revealed that complementation with *clbA* or *entD* allowed the survival of strain SP15 *entD clbA in vivo*, in a statistically significant manner at least in blood (Fig. 7B).

This evidenced that the presence of either functional EntD or ClbA is required to maintain full virulence of ExPEC in a mouse model of sepsis.

## Discussion

Our work demonstrates the interplay between the biosynthetic pathways of a genotoxin and multiple siderophores. We have shown that ClbA, encoded by the *pks* island, is a promiscuous PPTase which promotes the synthesis of colibactin, yersiniabactin, enterobactin and consequently salmochelins. Although we demonstrated that ClbA could substitute for an *entD* mutation, the reciprocity was not observed. EntD seems to be specific for the synthesis of siderophores, which is consistent with other published reports [32]. In contrast, YbtD, the PPTase involved in yersiniabactin production in *Yersinia* was shown to substitute for a *clbA* mutation and allowed the production of colibactin. Attempts to relate conserved motifs of the group II subfamily of PPTases [12] with substrate specificity did not allow us to understand the functional promiscuity evidenced among certain PPTases, since type II PPTases usually have very remote primary sequences. Unfortunately, it is not possible to compare either the 3D structure of these PPTases because only the structure of Sfp is available [33]. Type II PPTases are predicted to have a similar folding and very similar secondary structures [33]. However it is difficult to draw conclusions on the folding of proteins and to correlate it with substrate specificity. Only the comparison of 3D high-resolution structures would provide information about the structure/function relationship of PPTases. Our work provides novel evidence that make PPTases promising targets for antibacterial development [34], because these enzymes are crucial for the biosynthesis of a multitude of a pathogen's collection of essential metabolites and virulence factors [35].

Iron is an essential element for survival of *E. coli*. Therefore, *E. coli* strains have evolved a strategy for iron acquisition which uses multiple siderophores with high-affinity for ferric iron. These include enterobactin, salmochelins, aerobactin and yersiniabactin [11]. Each siderophore has specific affinity for iron and may be differentially regulated to provide different advantages, potentially allowing extra-intestinal pathogenic *E. coli* (ExPEC) to adapt to different environmental conditions or to overcome host innate immunity [10], [36], [37]. In our model of sepsis, the ExPEC mutant that produced only aerobactin as a siderophore (strain SP15 *entD clbA*) was completely attenuated. This suggests that aerobactin plays a minor role in the iron uptake in this sepsis model; but the importance of each siderophore can be host and strain dependent [38]. Interestingly, either ClbA or EntD were able to restore the virulence of strain SP15 *entD clbA*. However, we have shown that colibactin synthesis cannot be sustained by EntD. This suggests that not colibactin, but the siderophore systems (alone or in combination) are critical during the first step of the infection in this mouse model of sepsis. Indeed, the bacterial loads in both spleen and blood were similar in animal infected with SP15 *entD clbA* mutant complemented either with ClbA or EntD. Analysis of bacteria present in the popliteal lymph node confirmed this analysis (data not shown). Since the carriage of the *pks* island is correlated with successful long-term gut colonization in humans [39], colibactin could be important for the commensal lifestyle of ExPEC. Moreover, our unpublished data suggest that the genotoxin colibactin could also play a role in natural sepsis since lymphocytes are susceptible to the genotoxin.

**Figure 8:**
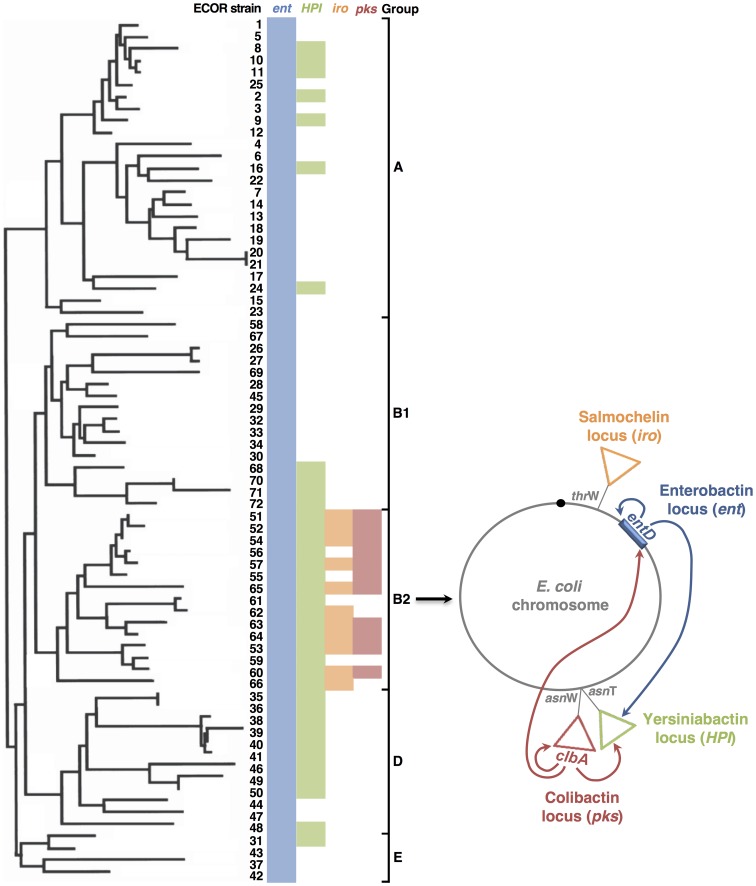
Model for the co-evolution of the *pks* and high pathogenicity islands in *E. coli*. **Left.** Phylogenetic relationships amongst the *E. coli* reference strains (ECOR, [3], [52]). The phylogeny was based on MLST of back-bone genes [3], [43]. Ent locus positive strains are indicated in blue, HPI island positive strains are indicated in green [43], iro locus positive strains are indicated in orange and *pks* island positive strains are indicated in pink [21]. The presence of the iro locus was determined only in B2 strains. **Right.** The archetypal chromosome of phylogroup B2 *E. coli* strains. The loci encoding enterobactin (ent), yersiniabactin (HPI), salmochelin (iro) and colibactin (pks) are represented. The arrows originating from PPTases EntD and ClbA and pointing towards other loci illustrate the capacity of the PPTase to contribute to the synthesis of metabolites from other loci.

Phylogroup B2, which includes the majority of ExPEC isolates, is considered to represent the evolutionary eldest lineage within the species [40]. Interestingly, the *pks* island found in B2 isolates is highly conserved, and is physically associated to a highly conserved High-Pathogenicity Island. This might even point towards a recent emergence of a distinct subgroup within phylogroup B2. In fact, epidemiological knowledge allows defining specific clonal lineages with high ExPEC virulence potential [41]. We believe that the most virulent and also the best colonizer of human gut resulted from a step-by-step acquisition and selection of different mobile elements. We propose here a scenario with the sequential integration of at least two pathogenicity islands and the cross talk via two PPTases (Fig. 8). At first, all *E. coli* strains produce at least one siderophore *i.e.* enterobactin. The *entD* gene and the other genes of the enterobactin system are part of the core genome and have been identified in all the *E. coli* strains isolated so far [42]. In contrast, the HPI encoding the yersiniabactin siderophore system devoid of any PPTase gene was acquired by horizontal gene transfer. Almost all *E. coli* HPIs appear to result from a single ancestor, which entered the *E. coli* species rather recently [43]. All strains of the phylogenetic group B2 and almost all of group D carry the HPI, whereas strains of groups A and B1 were found to be only occasionally HPI positive (Fig. 8, [19]). The spread of the HPI must have occurred in a dramatically fast fashion, which may indicate a strong selective pressure. We have shown in this study that EntD is actually the PPTase that mediates the synthesis of a functional yersiniabactin. *E. coli* strains that contain the HPI were demonstrated to be more virulent than isolates that lack the island [18]. Moreover, yersiniabactin is frequently associated with urinary tract infections [44], [45]. The *pks* island is known to be confined to the phylogenetic group B2. Besides, the *pks* island is highly represented within an especially highly virulent subset of B2 strains that exhibit extremely elevated virulence scores and an increased likelihood of causing bacteremia [25]. It has been previously demonstrated that all the *E. coli* strains that acquired the *pks* island encoding the colibactin through horizontal transfer, also displayed the HPI locus, with an integration site in tRNA *asnW* gene and *asnT* gene, respectively (Fig. 8; [26]). The *pks* island appears to be highly conserved (or even identical) in terms of nucleotide sequence in different *E. coli* isolates [26]. This may be a hint to a more recent acquisition of the *pks* island, compared to the HPI, which displays about 1–2% sequence divergence among the *E. coli* isolates (Schubert, unpublished data). We hypothesize that the association of the *pks* island with HPI has been selected in the highly virulent *E. coli* isolates because ClbA can contribute to the synthesis of both the genotoxin and yersiniabactin (and also enterobactin and consequently salmochelins). This deadly association is not confined in *E. coli*. Similar events also occurred in other pathogenic *Enterobacteriacae* since the *pks* island was also detected in *Klebsiella pneumoniae*, *Enterobacter aerogenes*, and *Citrobacter koseri* isolates where the island is also physically associated on the chromosome with the HPI locus [26].

## Materials and Methods

### Bacterial strains, mutagenesis procedures and growth conditions

Bacterial strains used in this study are listed in Table 1. *E. coli* SE15 (O150:H5) is a human commensal bacterium isolated from feces of a healthy adult and classified into *E. coli* phylogenetic group B2 [46]. Strain SE15 is devoid of the *pks* island. *E. coli* M1/5 is a human commensal bacterium isolated from feces of a healthy adult and classified into *E. coli* phylogenetic group B2. Strain M1/5 harbors of the *pks* island. Strain SP15 is an extra-intestinal pathogenic *E. coli* strain (ExPEC) of serotype O18:K1:H7 isolated from neonatal meningitis. Strain SP15 harbors the *pks* island. The repertoire of siderophores the *E. coli* strains possess is indicated in Table 1. Gene inactivations were engineered by using the lambda Red recombinase method [47] using primers listed in Table 2. For complementation, the *clbA* gene was cloned into plasmid pASK75, a cloning vector that harbors a pBR322 origin of replication and therefore is low copy number plasmid (p-*clbA* (1), table 1) or PCR-Script, a cloning vector that harbors a ColE1 origin of replication and therefore is high copy number plasmid (p-*clbA* (2), table 1). For complementation, the *entD* gene was cloned into PCR-Script (p-*entD*, table 1).

**Table 1:** Strains and plasmids used in the study.

Strain or plasmid	Genotype or phenotype	Source or reference
*E. coli* strains		
DH10B	Enterobactin siderophore producer	
DH5α	Enterobactin siderophore producer	
HB101	Enterobactin siderophore producer	
MG1655	Enterobactin siderophore producer	
WR1542+pACYC5.3L	Tc^r^, Ap^r^, Kan^r^, Cm^r^; *fepA*::Tn*10d*Tc, *iroN*::pGP704, *cir*::MudJ carrying pACYC5.3L plasmid	Gift from W. Rabsch
MG1655 *entE*	*entE* mutant of strain MG1655; Kan^r^	This study
MG1655 *entE*+BAC *pks*+	*entE* mutant of strain MG1655 carrying BAC *pks*+; Kan^r^, Cm^r^	This study
MG1655 *entD*	*entD* mutant of strain MG1655; Kan^r^	This study
MG1655 *entD*+BAC *pks*+	*entD* mutant of strain MG1655 carrying BAC *pks*+; Kan^r^, Cm^r^	This study
MG1655 *entD*+BAC *pks*Δ*clbA*	*entD* mutant of strain MG1655 carrying BAC *pks*Δ*clbA*; Kan^r^, Cm^r^	This study
MG1655 *entD*+p-*clbA* (1)	*entD* mutant of strain MG1655 carrying p-*clbA* (1); Kan^r^ Amp^r^	This study
MG1655 Δ*entD*	*entD* mutant of strain MG1655	This study
MG1655 Δ*entD*+p-*bpsA*	*entD* mutant of strain MG1655 carrying p-*bpsA*; Kan^r^	This study
MG1655 Δ*entD*+p-bpsA+p-*entD*	*entD* mutant of strain MG1655 carrying p-*bpsA* and p-*entD*; Kan^r^ Amp^r^	This study
MG1655 Δ*entD*+p-*bpsA*−+p-*clbA* (1)	*entD* mutant of strain MG1655 carrying p-*bpsA* and p-*clbA* (1); Kan^r^ Amp^r^	This study
MG1655 Δ*entD*+p-*bpsA*+p-*sfp*	*entD* mutant of strain MG1655 carrying p-*bpsA* and p-*sfp*; Kan^r^ Amp^r^	This study
MG1655 Δ*entD*+p-*bpsA*+p-*pptT*	*entD* mutant of strain MG1655 carrying p-*bpsA* and p-*pptT*; Kan^r^ Amp^r^	This study
MG1655+BAC *pks*+	strain MG1655 carrying BAC *pks*+; Cm^r^	This study
MG1655+BAC *pks*Δ*clbA*	strain MG1655 carrying BAC *pks*Δ*clbA*; Cm^r^	This study
MG1655+BAC *pks*++p-*bpsA*	strain MG1655 carrying BAC *pks*+ and p-*bpsA*; Kan^r^ Cm^r^	This study
MG1655+BAC *pks*Δ*clbA*+p-*bpsA*	strain MG1655 carrying BAC *pks*Δ*clbA* and p-*bpsA*; Kan^r^ Cm^r^	This study
SE15	Enterobactin and yersiniabactin siderophores producer	[46]
SE15 *entD*	*entD* mutant of strain SE15; Kan^r^	This study
SE15 *entD*+p-*entD*	*entD* mutant of strain SE15 carrying p-*entD* plasmid; Kan^r^ Amp^r^	This study
SE15 *entD*+p-*clbA* (1)	*entD* mutant of strain SE15 carrying p-*clbA* (1) plasmid; Kan^r^ Amp^r^	This study
M1/5	Enterobactin, aerobactin and yersiniabactin siderophores producer	Gift from U. Dobrindt
M1/5 *entD*	*entD* mutant of strain M1/5; Kan^r^	This study
M1/5 *clbA*	*clbA* mutant of strain M1/5; Kan^r^	This study
M1/5 *entD clbA*	*entD clbA* mutant of strain M1/5; Kan^r^	This study
M1/5 *entD clbA*+p-*entD*	*entD clbA* mutant of strain M1/5 carrying p-*entD*; Kan^r^ Amp^r^	This study
M1/5 *entD clbA*+p-*clbA* (1)	*entD clbA* mutant of strain M1/5 carrying p-*clbA* (1); Kan^r^ Amp^r^	This study
M1/5 *entD clbA*+p-*clbA* (2)	*entD clbA* mutant of strain M1/5 carrying p-*clbA* (2); Kan^r^ Amp^r^	This study
M1/5 *clbA*+p-*clbA* (1)	*clbA* mutant of strain M1/5 carrying p-*clbA* (1); Kan^r^ Amp^r^	This study
M1/5 *clbA*+p-*sfp*	*clbA* mutant of strain M1/5 carrying p-*sfp* plasmid; Kan^r^ Amp^r^	This study
M1/5 *clbA*+p-*pptT*	*clbA* mutant of strain M1/5 carrying p-*pptT* plasmid; Kan^r^ Amp^r^	This study
SP15	Enterobactin, salmochelin, aerobactin and yersiniabactin siderophores producer	[53]
SP15 *clbA*	*clbA* mutant of strain SP15; Kan^r^	This study
SP15 *entD*	*entD* mutant of strain SP15; Kan^r^	This study
SP15 *entD clbA*	*entD clbA* mutant of strain SP15; Kan^r^	This study
SP15 *entD clbA*+p-*clbA* (2)	*clbA* mutant of strain SP15 carrying p-*clbA* (2); Kan^r^ Amp^r^	This study
SP15 *entD clbA*+p-*entD*	*entD clbA* mutant of strain SP15 carrying p-*entD*; Kan^r^ Amp^r^	This study
Plasmids		
pACYC5.3L	*fyuA*-, *ybtA*-, *fyuA-luc*-, *irp6-8*, Cm^r^	Gift from W. Rabsch
p-*entD*	High copy number PCR-Script plasmid carrying *entD* gene; Amp^r^	This study
p-*clbA* (1)	Low copy number pASK75 plasmid carrying *clbA* gene; Amp^r^	Gift from U. Dobrindt
p-*clbA* (2)	pMB808, high copy number PCR-Script plasmid carrying *clbA* gene; Amp^r^	[22]
BAC *pks*+	Bacterial artificial chromosome carrying the entire *pks* island; Cm^r^	[21]
BAC *pks*Δ*clbA*	Bacterial artificial chromosome carrying the entire *pks* island with deleted *clbA* gene; Cm^r^	[22]
p-*sfp*	Low copy number pET26b plasmid carrying *sfp* gene from *Bacillus subtilis*; Amp^r^	Gift from C. Chalut
p-*pptT*	Low copy number pET28a plasmid carrying *pptT* gene from *Mycobacterium tuberculosis*; Amp^r^	Gift from C. Chalut
p-*bpsA*	Low copy number pET26b plasmid carrying gene *bpsA* from *Streptomyces lavendulae*, Kan^r^	Gift from C. Chalut

**Table 2:** Oligonucleotides used in the study.

Primers	Sequences
entD-P1	GGGCGGATCGCTGCAATTTATGCGCTGCGGGAATATGGCTATAAATGTGTGCGTGTAGGCTGGAGCTGCTTC
entD-P2	TCACTTGCCTTAAATGCGCTCTCTTTGGCGGAAAATGCCAGTGTCAGCGCCATATGAATATCCTCCTTAG
entD-Up	CCCCCGGGGGGGACGTACGTGGTATATGAGC
entD-Down	AACTGCAGAAGCACCTGCTTTACACTTTCG
entE-P1	TATCGACGGCGAGCGACAGTTGAGTTATCGGGAGCTGAATCAGGCGGCGTGTAGGCTGGAGCTGCTTC
entE-P2	AAGCGCAGCTTTTTTCGCCCATCAGCTCATCTTCCATGCTCACCAGTGCCATATGAATATCCTCCTTAG
JPN42	CAG ATA CAC AGA TAC CAT TCA
JPN46	CTA GAT TAT CCG TGG CGA TTC

Before injection to mice, all *E. coli* strains were grown overnight in LB broth supplemented with antibiotics if required, at 37°C with shaking. These cultures were diluted 1∶100 in LB broth with antibiotics when necessary and grown for 3 h at 37°C with shaking. Bacterial cells were resuspended in sterile PBS to the appropriate concentration (2×10^9^ CFU/mL). All the strains were shown to display similar growth kinetics *in vitro* in LB broth (data not shown).

### Detection and quantification of total siderophores

Chrome azurol S (CAS) assay was used to detect siderophores produced by *E. coli*. The CAS solution was prepared according to Schwyn and Neilands [48]. *E. coli* strains were grown on CAS agar plates and incubated at 37°C overnight in the dark. The colonies with orange zones were siderophore-producing strains [48].

To quantify siderophore synthesis, 500 µL of CAS indicator solution containing 4 mM sulfosalicylic acid was mixed with the same volume of supernatant. The reaction mixtures were incubated for 60 min at room temperature to allow complex formation, and the siderophore-dependent color change was determined at OD_630 nm_. For quantification, the iron chelating agent 8 hydroxyquinoline (8HQ, sigma-aldrich) was used as the standard.

### Quantification of yersiniabactin

The expression of the *fyuA* gene encoding the yersiniabactin receptor (FyuA) is known to be up-regulated in the presence of extracellular yersiniabactin [49]. Thus, yersiniabactin-dependent up-regulation of *fyuA* expression can be monitored by means of a *fyuA*-reporter fusion in the indicator strain [50].

Bacterial strains were cultivated in NBD medium, *i.e.* Nutrient Broth (NB) medium supplemented with 200 µM α,α'-dipyridyl (Sigma), for 24 h at 37°C. Bacteria were pelleted by centrifugation and the supernatant was added to the indicator strain WR1542 carrying plasmid pACYC5.3L (kind gift of W. Rabsch, Wernigerode). The plasmid encodes all genes necessary for yersiniabactin uptake; *i.e. irp6*, *irp7*, *irp8*, *fyuA* and *ybtA*. Additionally, the *fyuA* promoter region fused to the luciferase reporter gene is included on pACYC5.3L. After further 24 h of incubation at 37°C the indicator strain was pelleted and resuspended in bacterial lysis buffer (100 mM potassium phosphate buffer [pH 7.8], 2 mM EDTA, 1% [wt/vol] Triton X-100, 5 mg/ml bovine serum albumin, 1 mM dithiothreitol, 5 mg/ml lysozyme). Complete lysis was performed by incubation at room temperature for 20 min and repeated mixing. The samples were centrifuged and supernatants of lysates were analyzed by addition of luciferase reagent (20 mM Tricine-HCl (pH 7.8), 1.07 mM (MgCO_3_)_4_ Mg(OH)_2_, 100 µM EDTA, 470 µM D(-) luciferin, 33.3 mM dithiothreitol, 270 µM Li_3_ coenzyme A, 530 µM Mg-ATP). Luciferase activities were determined in triplicates using the multimode reader Berthold Tristar LB 941. Values were corrected by relating luciferase activity to the OD_600_ of bacterial cultures grown 24 h in NBD medium. K12 *E. coli* strain DH5α served as negative control. The experiments were repeated at least three times.

### Detection and quantification of indigoidine

After overnight cultures in LB broth supplemented with the appropriate antibiotics, bacteria were diluted 1∶10 in M9 minimal medium supplemented with 100 mM L-glutamine and 1 mM IPTG, and cultivated 16 h at 18–20°C under shaking [31], [51]. Bacteria were then collected by centrifugation at 900× *g* for 5 min. At this speed, bacterial cells were pelleted while indigoidine still remained in the supernatant [51]. Indigoidine production was quantified by measuring the absorbance of blue-colored supernatant (OD_612 nm_). The bacterial pellet was resuspended in PBS, and biomass was quantified by measuring the absorbance (OD_450 nm_). Finally, the indigoidine production was normalized with the ratio Indigoidine/Biomass (*e.g.* ratio OD_612 nm_/OD_450 nm_).

### Determination of the megalocytosis induced by colibactin

HeLa cells were maintained by serial passage in DMEM supplemented with 10% FCS, non-essential amino acids and 50 µg/mL gentamicin. HeLa cells were dispensed in 96-well cell culture plate (5×10^3^ cells/well). For bacterial infections, overnight LB broth cultures of *E. coli* were diluted in interaction medium (DMEM, 5% FCS, 25 mM HEPES) and cell cultures (∼70% confluent) were infected with a multiplicity of infection (number of bacteria per HeLa cell at the onset of infection) of 3 to 400. Four hours post-inoculation, cells were washed 3 times with HBSS and incubated in cell culture medium 72 h with 200 µg/mL gentamicin before protein staining with methylene blue (1% w/v in Tris-HCl 0.01M). The methylene blue was extracted with HCl 0.1N. The quantification of staining was measured at OD_660 nm_.

### Determination of the genotoxic effect induced by colibactin

The In Cell Western procedure was performed as described previously [27]. Briefly, HeLa cells were dispensed in 96-well cell culture plate (1.5×10^5^ cells/200 µL/wells). Twenty four hours later, cells were infected with *E. coli* strains for 4 h. Eight hours post-infection the cells were directly fixed in the plate with 4% paraformaldehyde. Paraformaldehyde was neutralized, and cells were permeabilized as previously described [27]. Cells were blocked with MAXblock Blocking medium (Active Motif, Belgium) supplemented with phosphatase inhibitor PHOSTOP (Roche), followed by overnight incubation with rabbit monoclonal anti γ-H2AX (Cell Signaling) (1∶200). An infrared fluorescent secondary antibody absorbing at 800 nm (IRDyeTM 800CW, Rockland) was then applied (dilution 1∶500). For DNA labeling, RedDot2 (Biotium) was used (dilution 1∶500) together with the secondary antibody. The DNA and the γ-H2AX were simultaneously visualized using an Odyssey Infrared Imaging Scanner (Li-Cor ScienceTec, Les Ulis, France) with the 680 nm fluorophore (red color) and the 800 nm fluorophore (green dye). Relative fluorescence units from the scanning allowed a quantitative analysis. Relative fluorescent units for γ-H2AX per cell (as determined by γ-H2AX divided by DNA content) were divided by vehicle controls to determine percent change in phosphorylation of H2AX levels relative to control. All experiments were carried out in triplicate.

### Mouse sepsis model

Animal experimentations were carried out in accordance with the European directive for the protection of animals used for scientific purposes. The protocols were validated by the local ethics committee on animal experiment “Comité d'éthique Midi Pyrénées pour l'expérimentation animale” which is affiliated to “Comité National de Réflexion Ethique sur l'Expérimentation Animale” and linked to the french ministry of research (Referenced protocols: PX-ANI-A2-94, 95, 96, 99, 100, and 101). Nine week old female C57BL/6J mice (JANVIER) were injected into the footpad with 10^8^ ExPEC WT, *clbA* mutants, *entD* mutant, *entD clbA* mutant and *entD clbA* mutant complemented with *clbA* (*entD clbA*+p-*clbA*(2)) and *entD* (*entD clbA*+p-*entD*), together with intraperitoneal injection of 100 µL of carbenicillin (1.6 mg/mL) or PBS. When required, mice were sacrificed by lethal anaesthesia (rompun/ketamine in 0.9% NaCl) 18 h post injection. The abdominal cavity of anesthetized mouse was opened. The widest part of the posterior vena cava was localized and sectioned. Blood was collected by aspiration from the abdominal cavity. Spleens were surgically removed. Bacteria located in spleen cells were isolated from the mechanical dissociation of the splenic tissue using Precellys tissue homogenizer. Bacteria were quantified by plating of serial dilutions of blood and dissociated spleen on appropriate selective MacConkey agar. The antibiotics used to supplement the medium correspond to the resistance displayed by the different strains and are indicated in Table 1.

### Statistical analysis

Statistical analyses were conducted using GraphPad Prism 5.0d. The mean ± standard deviation (SD) is shown in figures, and *P* values were calculated using a one-way or two-way ANOVA followed by a Bonferroni post-test unless otherwise stated. For bacterial quantification, CFU by mg of spleen or mL of blood were log transformed for the analysis. A *P* value of less than 0.05 was considered statistically significant and is denoted by *. *P*<0.01 is denoted by ** and *P*<0.001 by ***.
